# Bioengineering Technologies for Cardiac Regenerative Medicine

**DOI:** 10.3389/fbioe.2021.681705

**Published:** 2021-06-03

**Authors:** Mira Chingale, Dashuai Zhu, Ke Cheng, Ke Huang

**Affiliations:** ^1^Department of Molecular Biomedical Sciences and Comparative Medicine Institute, North Carolina State University, Raleigh, NC, United States; ^2^Joint Department of Biomedical Engineering, University of North Carolina at Chapel Hill, North Carolina State University, Raleigh, NC, United States

**Keywords:** bioengineering, cardiac repair, cell reprogramming, exosome, cardiac patch, targeting

## Abstract

Cardiac regenerative medicine faces big challenges such as a lack of adult cardiac stem cells, low turnover of mature cardiomyocytes, and difficulty in therapeutic delivery to the injured heart. The interaction of bioengineering and cardiac regenerative medicine offers innovative solutions to this field. For example, cell reprogramming technology has been applied by both direct and indirect routes to generate patient-specific cardiomyocytes. Various viral and non-viral vectors have been utilized for gene editing to intervene gene expression patterns during the cardiac remodeling process. Cell-derived protein factors, exosomes, and miRNAs have been isolated and delivered through engineered particles to overcome many innate limitations of live cell therapy. Protein decoration, antibody modification, and platelet membranes have been used for targeting and precision medicine. Cardiac patches have been used for transferring therapeutics with better retention and integration. Other technologies such as 3D printing and 3D culture have been used to create replaceable cardiac tissue. In this review, we discuss recent advancements in bioengineering and biotechnologies for cardiac regenerative medicine.

## Introduction

Cardiovascular disease (CVD) is the leading cause of death in both developed and developing countries. According to the World Health Organization (WHO), 17.9 million people across the globe (31%) die due to CVD, of which 85% die from myocardial infarction (MI) (World Health Organization, 2021). In adult hearts post-MI, injured heart muscle cells are replaced by fibrotic tissue. During this maladaptive remodeling process, activated cardiac fibroblasts turn into myofibroblasts, causing stiffness and fibrosis, which is, in turn, associated with poor prognosis and heart failure. Though cardiac fibroblasts provide structural integrity to the heart after MI, it also causes a non-contracting scar. Such events hamper cardiac perfusion and pumping capacity and leads to cardiac remodeling further toward depravation to cardiac dysfunction, myocardium loss, and, eventually, heart failure (Xin et al., 2013; Saparov et al., 2017; Tallquist and Molkentin, 2017; Bo et al., 2020; Nelson and Brunt, 2021).

Conventional treatments for MI include coronary artery bypass, coronary reperfusion therapy, and fibrinolytic therapy, which are mainly for acute symptom relief rather than to promote repair and regeneration of the damaged myocardium (Awada et al., 2015). A heart transplant or a left ventricular assist device (LVAD) (Rose et al., 2001) is the final treatment option for heart failure patients. However, prognosis varies due to the complexity of the required highly invasive transplant surgery and its subsequent acute/chronic immune rejections (White and Chew, 2008; Wilhelm, 2015). Although pharmacological treatments of β-blockers and angiotensin-converting enzyme (ACE) inhibitors (Packer et al., 2001; McMurray et al., 2014) are beneficial to MI patients, these existing approaches make it necessary to explore new methods of treatment that aim at regenerating the infarcted myocardium as well as becoming implementable in the clinical practices (Raziyeva et al., 2020).

**FIGURE 1:**
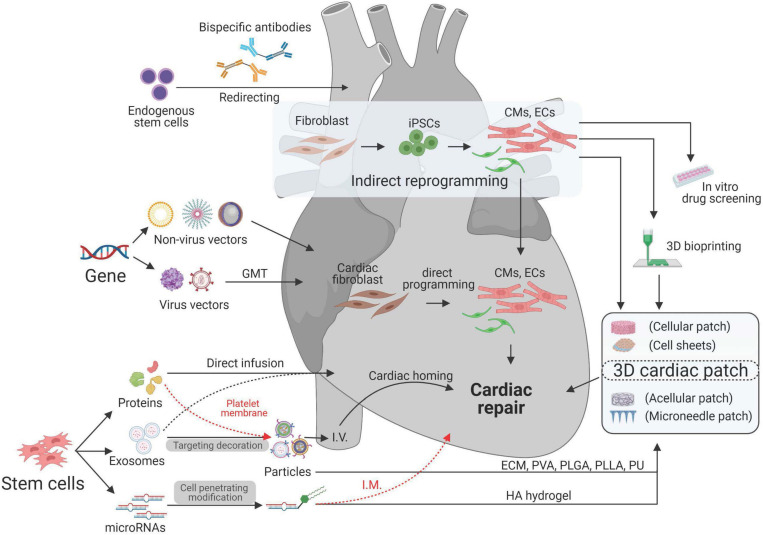
Bioengineering and biotechnologies for cardiac repair.

Biomedical engineering seeks to close the gap between engineering and medicine by combining the design and problem-solving skills of engineering with medical and biological sciences. Biomedical engineering hopes to advance health care treatment, including diagnosis, monitoring, and therapy. It has been transforming the cardiac regenerative approaches into potential treatments for CVD (Lee and Walsh, 2016). Treatments for ischemic/reperfusion-damaged or infarcted myocardium have been designed by using multifarious biotechnologies based on the purpose of treatment. With the idea of using autologous cells for cardiac treatment, patient-specific cardiomyocytes (CMs) were generated through cell reprogramming technologies (Wang et al., 2021). To improve the regenerative capability of CMs, various viral and non-viral vectors have been used for gene editing to intervene with gene expression during cardiac remodeling process after MI (Rincon et al., 2015; Kohama et al., 2020). To overcome the retention, fragile, tumorigenicity, and immunogenicity limitations of cell therapy (Tang et al., 2018c), cell-derived protein factors, exosomes, and miRNAs have been isolated and delivered through micro- (Huang et al., 2020) or nanosized particles (Su et al., 2018b). For better targeting, scientists have used proteins, antibodies, and platelet membranes to decorate their therapeutics. For better retention and integration, cardiac patches have been designed by transfer therapeutics in vehicles made of various biomaterials (Mei and Cheng, 2020). Additionally, 3D printing (Maiullari et al., 2018) and 3D culture (Jackman et al., 2018) technologies were utilized to create replaceable cardiac tissue (Figure 1 and Table 1). In this review, we will discuss current biotechnologies for cardiac repair.

**TABLE 1:** Summarization of advantages and disadvantages in different bioengineering methods.

Bioengineered methods	Advantages	Disadvantages
Direct cell reprogramming	• Needs no iPSC stage • Possibility for *in situ* reprogramming • Relatively rapid conversion	• Low efficiency • Unclear cell fate stability • Tumorigenicity risks
Indirect cell reprogramming	• High efficiency • iPSC induction from many cell types • Self-renewal of iPSCs • Differentiation condition is modulable • Cell fate stability • Suitable for *ex vivo* manipulations	• Requires iPSC stage • Only can be performed *in vitro* • The CMs are not mature • Tumorigenicity risks
Non-viral gene delivery	• Non-pathogenic • Multiple delivery methods. e.g., Naked DNA direct delivery, gene gun, electroporation, ultrasound, lipoplexes and polyplexes etc. • Simplicity in manufacturing • Flexibility in packaging capacity	• Low transduction efficiency • Cytotoxicity • Transient transfection • Tissue damage • Shallow penetration • Immunogenicity
Viral gene delivery	• High transduction efficiency • Transient or persistent expression • Multiple viral vector choices	• Difficult to produce high viral titers • Immunogenicity • Limitations in gene packaging capacity • Safety issues • Expensive •
Synthetic particles	• High biocompatibility • Cryo-stability • Easy manipulation of particle contents • Cardiac targeting can be achieved • Degradable • Multiple delivery routes • Controlled release of therapeutics	• Hard to control the size and morphology • Requires multiple fabrication steps • Low encapsulation efficiency • Sensitive to operational parameters • Mostly paracrine effects, lack of mechanism to generate new CMs
Natural particles (extracellular vesicles and exosomes)	• High biocompatibility • Low immunogenicity • Low tumorigeneicity • Long term stability	• Quality depending on cell batches and culture condition • Variation in batches • Low yield and purity • Difficulty in isolation
Non-cellular cardiac patches	• Multiple biomaterial selections • High biocompatibility • Easy manufacturing and off-the-shelf • Suitable for large amount of cargo delivery • Easy storage and transfer	• Hard to transplant *in vivo* (open-chest surgery is required) • Low cardiac integration • Low cardiac penetration •
Cellular cardiac patches	• Highly biocompatible • Highly functional • Suitable for both *in vivo* and *in vitro* studies • Multiple designs	• Tumorigenicity • The maturation of CMs in tissue patch • Hard to manufacture • Batch-to-batch variation (depending on cell quality) • Very fragile and hard to transfer or storage • Low vascularization after transplantation • Arrhythmia risks after transplantation

## Cell Reprogramming

Cell reprogramming is a powerful tool that converts the somatic cell lineage into pluripotent stem cells (iPSCs) (Rao and Malik, 2012), CMs (Fu et al., 2015) or endothelial cells (ECs) (Lee C. S. et al., 2017). Generally, this tool is used both *in vitro* and *in vivo* for cardiac injury site repair (Patel et al., 2016), cardiac disease modeling, or drug screening (Ebert et al., 2012; Chen and Vunjak-Novakovic, 2018). In the process of changing cell fate, an intermediary pluripotent state is key to differentiate direct and indirect reprogramming (Wang et al., 2021).

### Indirect Cell Reprogramming

Indirect cell reprogramming from adult somatic cells to iPSC-derived CMs (iPSC-CMs) is a well-established process (Tai et al., 2018). iPSCs from multiple origins are now commercially available. This reprogramming method is widely used not only due to the difficulty of culturing human primary CMs *in vitro* but also because they contain patient-specific genomic information and could be used for autologous cardiac regenerative medicine (Martins et al., 2014). Commonly, adult fibroblasts are reprogrammed into iPSCs through the activation of alkaline phosphatase, silencing of somatic-specific expression, expression of SSEA1, and progressive silencing of exogenous genes with upregulation of Oct4 and Nanog (Teshigawara et al., 2017). However, these CMs are closer to an immature stage in terms of marker expression, ultrastructural features, metabolic signature, and electrophysiological properties (Tang, 2020). First, the origin of somatic cells is a determinant of iPSC-CM maturation (Pianezzi et al., 2020). Comparison of iPSC-CMs derived from cardiac-derived mesenchymal progenitor cells (CPCs), bone marrow-derived mesenchymal stem cells (BMCs), and human dermal fibroblasts (HDFs) that comes from the same patient showed the cardiac somatic cell’s enhanced capacity for cardiac re-differentiation due to upregulated cardiac genes (*MYH6, TNNI3, KCNQ1, KCNE1*) (Altomare et al., 2016; Pianezzi et al., 2020). Additionally, the application of iPSC-CMs is highly affected by the purification process because tumors can form during *in vitro* culture of iPSCs that increase the malignant risks to *in vivo* application (Tohyama et al., 2013). To overcome the purification obstacles, a distinct metabolic flow technology has been designed to enable large-scale purification through glucose depletion and lactate supplementation because the mature iPSC-CMs have a higher oxygen consumption rate with increased mitochondrial maturity (Tohyama et al., 2013; Tang, 2020).

### Direct Cell Reprogramming

Direct cell reprograming is a process of transforming of somatic cells to a desired cell fate without a pluripotent or multipotent state (Wang et al., 2021). Ideally, direct cell reprogramming is more suitable for *in vivo* cardiac tissue repair by generating reprogrammed cells *in situ* in the diseased heart (Wang et al., 2021); however, it is still challenging to perform it *in vivo* due to the low transforming efficiency. For example, direct reprogramming of transcriptional factors like Gata4, Oct4, Tbx5, Sox2, and Klf4 were delivered directly into the damaged heart to initiate regeneration (Ieda et al., 2010; Chen et al., 2017; Hashimoto et al., 2018). Six core transcriptional factors Gata4, Hand2, Mef2c, Mesp1, Nkx2.5, and Tbx5 were examined for their cardiac linage reprograming capability (Li et al., 2015; Wang et al., 2015). Retroviruses were used to express these transcription factors in fibroblasts that were derived from adult mice (Pasumarthi and Field, 2002; Ieda et al., 2010; Song et al., 2012). Another study reported *in vitro* formation of CMs from fibroblasts by expressing transcriptional factors Gata4, Mef2c, and Tbx5 (GMT) and thereby functionally repopulating the scar (Qian et al., 2012; Wang et al., 2015). Although direct cell reprogramming bypasses early developmental stages (Barreto et al., 2019) such as the cardiac progenitor stages, the tumorigenic risks may not lower than indirect reprogramming because the small molecules cannot be guaranteed, which can also produce iPSCs (Chen et al., 2017). Most importantly, the fate of transduced cells *in vivo* is still debated, although single-cell transcriptomics have been done to discover the mechanism of the fate conversion from fibroblast to CMs (Liu et al., 2017). Additionally, miRNAs have the ability to regulate various signaling pathways at the same time, which makes them a promising alternative (Sandmaier and Telugu, 2015). Researchers provided evidence that direct administration of miRNAs through lipid-based transfection at the target site successfully converted fibroblasts into cardiomyocytes *in vivo* (Elmén et al., 2008; Sridharan and Plath, 2011). Cardiac reprogramming through miRNAs (miR-1 miR-133, miR208, and miR499) was enhanced when combined with JAK inhibitor I treatment (Jayawardena et al., 2012).

## Gene Editing

Many genes are essential for CM proliferation and cardiac repair. For example, ERBB2 has been reported triggering mammalian heart regeneration by promoting cardiomyocyte dedifferentiation and proliferation through YAP activation and EMT (epithelial–mesenchymal transition)-like processes (D’Uva et al., 2015; Aharonov et al., 2020). Cyclin A2 or CCNA2, a gene normally silenced after birth, has been demonstrated as a key cell cycle regulatory gene to induce cardiac repair by mediating both the G1–S and G2–M transitions of the cell cycle (Shapiro et al., 2014). So, an efficient delivery of the desired gene to heart is important for cardiac gene therapy.

### Non-viral Gene Delivery

In cardiac gene therapy, efficient delivery of the desired gene to target tissue is important. Non-viral gene delivery methods, such as needle or jet injection, hydrodynamic gene transfer, electroporation, and cationic lipids make use of natural or synthetic compounds or physical forces to deliver the gene of interest to the target site. It is an important technology for tissue engineering with many features, including low toxicity, easy modification, high productivity, and cell specificity (Wu et al., 2018). A needleless liquid jet methodology using a jet device with micro jets has been used to expand or generate cell pores for cardiac gene delivery. However, it is not suitable for cardiac applications due to the jet’s piercing with high force (Fargnoli et al., 2017). Additionally, hydrophilic naked DNA could be consumed by cells (Al-Dosari and Gao, 2009); however, only a small percentage of target cells express the delivered genes, thus, making them inefficient.

### Viral Gene Delivery

Recombinant viral vectors such as adenoviruses, lentiviruses, and adeno-associated viruses are generally used for gene delivery (Wolfram and Donahue, 2013). Adenoviral gene delivery systems have been extensively used in gene-based therapies and cell-based therapies. Adenoviral vectors have shown a high transduction rate in both dividing and non-dividing cells (Lee S. et al., 2017). The knockdown of lncRNA through adenovirus-mediated lncRNA approaches lead to the reduction in macrophage infiltration and cardiomyocyte apoptosis in the heart after MI (Wang K. et al., 2017). Although adenovirus vectors were used in clinical trials due to their large tropism profile (Jessup et al., 2011), adenoviral-mediated gene delivery triggers immunogenicity in humans and lacks integration with the host genome, making it an unfavorable choice (Vilaysane and Muruve, 2009; Wasala et al., 2011). Lentiviral vectors are suitable for long-term transgene expression, which integrates their genome into the hosts with a preference for transcriptionally active sites (Bulcha et al., 2021). Lentiviral vectors can also elicit relatively weak immune responses through vector design (Bulcha et al., 2021). Lentiviral-mediated CCND2 gene transfection enhanced the regenerative potency of iPSCs-CMs (Zhu et al., 2018). Additionally, enhanced expression of Gata-4 and Nkz2.5 by lentiviral-mediated prodynorphin vectors resulted in a drastic increase in the CMs’ beating activity (Rincon et al., 2015). However, lentiviral vectors are limiting for cardiovascular disease treatments due to their low transduction rate in the myocardium *in vivo* (Rincon et al., 2015). The adeno-associated virus (AVV), as a cardiotropic vector, can be designed as a viral therapeutic to promote cardiac repair after MI (Yoo et al., 2018). AAV6 was reported as the most effective vector to transduce CMs (Ambrosi et al., 2019). For example, AAV6 was designed to deliver miR-199a to a pig model of MI (Gabisonia et al., 2019). Although the results showed cardiac improvement after 1 month, all pigs died due to subsequent uncontrolled expression of miR-199a that resulted in sudden arrhythmia.

## Particle Design

Stem cell therapy has been a promising approach mediated through paracrine effects (Maghin et al., 2020; Sid-Otmane et al., 2020). However, engraftment of transplanted cells and their low retention limit its therapeutic efficacy. Also, other concerns such as tumorigenicity, immunogenicity, and product stability need to be taken into account (Tang et al., 2018c). Since stem cells secrete a plethora of molecules like cytokines, soluble proteins, and extracellular vesicles that help in cardiac repair and regeneration (Tao et al., 2018), analysis of the cell secretome has gained importance in elucidating therapeutic mechanisms (Ellison-Hughes and Madeddu, 2017).

### Synthetic Particles

Micro-sized synthetic stem cells (SSCs) have been fabricated to mimic the paracrine effects of a live cell through a cell-mimicking microparticle (CMMP) technology (Tang et al., 2017; Huang et al., 2020). Briefly, poly lactic-co-glycolic acid (PLGA) is used to encapsulate the stem cell-derived secretomes. Afterward, microparticles are coated with cell membranes to become SSCs (Tang et al., 2017). This technology not only overcomes the multiple inherent limitations of live-cell therapy (Huang et al., 2020) but also holds a high applicability for different cell lines, for example, both cardiac stromal cells (CSCs) and mesenchymal stem cells (MSCs) have been synthetically created (Luo et al., 2017; Tang et al., 2017; Huang et al., 2020). Additionally, to transfer miR21 into macrophages and ECs, positively charged mesoporous silica nanoparticles (MSNs) are designed to encapsulate miR21 and are injected into the ischemic heart of pigs for treatment (Li Y. et al., 2021). MSNs are reported as a highly biocompatible and transfection-effective nanoparticle that are generated through a classical CTAB-templated, base-catalyzed sol–gel method (Li Y. et al., 2021). These microparticles exert their beneficial effects mainly through mimicking cell paracrine of protein factors and membrane-based cell–cell interaction with injured cells (Luo et al., 2017; Tang et al., 2017). Also, the nanoparticles that are consumed by CMs and fibroblasts release the enveloped miRNAs and proteins to regulate gene expression (Hodgkinson et al., 2015). To fully understand the mechanism, the combination of cell secretomes have to be further studied because the cardiac beneficial results may come from the combination or any of the secretome contents, including miRNAs, protein factors, exosomes, extracellular vesicles, and so on. Moreover, cell secretome is not standardized due to the variation of cell lines, culture conditions, and purity, which makes it even harder to uncover the veil of cell therapy.

### Natural Particles

Exosomes, a type of EVs, are bilipid layered nanovesicles with a diameter of around 35–150 nm that carry encapsulated proteins, membrane-bound proteins, and miRNAs and are capable of triggering various complex function-altering pathways (Li et al., 2017; Sun et al., 2018). Their small size allows them to pass through small capillaries, giving them more access to tissues than transplanted cells (Verweij et al., 2019; Dinh et al., 2020). Exosomes can be extracted and purified through techniques such as immunoaffinity capture, polymeric precipitation, tangential flow ultracentrifugation and size exclusion. Exosomes isolated from several cell lines including MSCs (Huang P. et al., 2019; Hu et al., 2021), iPSCs (Gao et al., 2020), cardiosphere-derived cells (CDCs) (Vandergriff et al., 2015; Gallet et al., 2017), and CPCs (Barile et al., 2017) have demonstrated cardiac protection through neovascularization and anti-inflammation (Teng et al., 2015; Huang P. et al., 2019). MSC-derived exosomes also contain miRNAs (miR-30b, let-7f, miR-424, and miR-30c) that promote angiogenesis For example, MSC-derived exosomal miR-21-5p heightens cardiac contractile strength and calcium handling through PI3K signaling (Mayourian et al., 2018; Qiao et al., 2019).

## Cardiac Targeting

Heart stem cell therapy is usually administered intramyocardially *via* open-chest or percutaneous coronary intervention (PCI) (Malliaras and Marbán, 2011). As the invasive nature of this drug delivery is not effective and appealing, researchers focus on developing therapeutics with targeting ability to the injured myocardium. This concept allows drugs to interact specifically with the infracted region and impart therapeutic benefits. Various targeting strategies, such as cardiotropic vector selection, peptide decoration, magnetic reactive carrier, or antibody editing have been employed for efficient drug delivery (Cores et al., 2015; Weinberger and Eschenhagen, 2021).

### Cardiotropic Vector Selection

Cardiac gene therapy requires viral vectors to safely access the heart specifically. So, it is essential to find a cardiotropic vector for gene delivery. For example, although multiple strains of AAVs from serotypes 1 to 9 have been isolated, only AAV9 (Inagaki et al., 2006) and AAV6 (Bish et al., 2008; Gao et al., 2011) showed a higher transduction efficiency. However, these vectors require intrapericardial or intramyocardial administration. Since there is a difference in host specificity (Asokan and Samulski, 2013), it is even harder to evaluate AAV vectors in preclinical models and clinical translation.

### Peptide Targeting

Peptide-based targeting has been achieved by decorating peptide (Kanki et al., 2011) onto therapeutics. A 12-amino acid non-naturally occurring peptide NH2-APWHLSSQYSRT-COOH was reported as a cardiomyocyte-targeting peptide (Feldman and Zahid, 2020). The peptide motif CSTSMLKAC was also identified as a potential tool for heart homing (Shelke et al., 2008; Kanki et al., 2011). For example, CDC-derived exosomes were designed to target the heart injury site *via* cardiac homing peptide CSTSMLKAC through a dioleoylphosphatidylethanolamine *N*-hydroxysuccinimide (DOPE-NHS) linker on the exosomal membrane (Shelke et al., 2008). Since the prostaglandin E2 (PGE2) receptors are overexpressed in the pathological cardiac microenvironment after ischemic/reperfusion injury (Kim et al., 2008; Zhang et al., 2015), researchers decorated PGE2 on nanoparticles through EDC/NHS coupling chemistry to increase cardiac homing capability (Su et al., 2018b).

### Platelet Targeting

Platelet-based targeting has been designed based on the inherent properties of platelets. Platelet surface receptors such as the GPIb/IX/V complex, GPVI and α2β1, β1, and β3 integrins, are responsible for interactions with exposed injury sites (Li et al., 2018). The platelet membrane has been isolated and coated on either cell (Shen et al., 2016; Tang et al., 2018a), micro- (Li et al., 2020) or nano- (Su et al., 2018b) particles to endow cardiac injury site homing capability (Li et al., 2018). To increase targeting and cellular uptake of nanoparticles on coronary artery stents, scientists activated ECs through binding P-selectin to platelet glycoprotein Ibα (GP Ibα) on platelet-mimicking nanoparticles (Lin et al., 2010).

### Antibody Targeting

There are many cardiac targeting approaches designed to utilize the innate specific binding capability of antibodies. Bispecific antibodies (BsAbs) have been designed to bind two different targets simultaneously by combining variable domains of desired monoclonal antibodies into an integrated structure (Huang et al., 2019a). BsAbs are generated by chemical conjugation, hybridoma fusion, or genetic engineering such as recombinant DNA technology (Parashar et al., 2011). To redirect endogenous bone marrow stem cells (BMSCs) to the injured heart, BsAbs were designed to link F(ab′)2 fragments from monoclonal anti-CD34 and anti-cardiac myosin heavy chain through chemical cycloaddition of AZ-PEG-NHS or DBCO-PEG-NHS on those F(ab′)2 fragments (Huang et al., 2019b). In this study, after G-CSF stimulation, the administration of BsAbs redirected circulating BMSCs to the injured myocardium (Huang et al., 2019b). Additionally, an inhalable platelet-targeting bispecific antibody (PT-BsAb) was designed by linking of CD34 (HSC binding) and CD42b (platelet binding) to redirect stem cells from the lungs to the heart for repair (Liu M. et al., 2021).

To increase the targeting capability of the injury site, a poly (*N*-isopropylacrylamide) nanogel with tissue plasminogen activator (tPA) and cell contractility inhibitor Y-27632 coupled anti-fibrin antibodies on the outside of nanoparticles through an EDC/sulfo-NHA method (Mihalko et al., 2018). The injected nanoparticles were directed by anti-fibrin antibodies to the fibrin-rich site post-MI and released tPA and Y-27632 to the site of injury (Mihalko et al., 2018; Huang et al., 2019b). Besides targeting, antibodies were also decorated on platelet-inspired cardiac-targeting microparticles to neutralize inflammatory cytokines. For example, since IL-1β has been demonstrated as a primary pro-inflammatory cytokine during cardiac remodeling, IL-1β antibodies were linked to platelet membrane on microparticles through 1,2-distearoyl-sn-glycero-3-phosphoethanolamine-poly (ethylene glycol) (DSPE-PEG) (Li et al., 2020).

## Cardiac Patch Design

Cardiac patches have been devised to ameliorate the cardiac function post-MI (Mei and Cheng, 2020). A cardiac patch is typically composed of substrate and active therapeutic agents (Yang et al., 2013). Cardiac patches can have therapeutic ingredients ranging from cells, such as iPSCs, myoblasts and MSCs, to bioactive molecules, such as miRNAs, exosomes, and microparticles (Mei et al., 2010; Squillaro et al., 2016; Wang L. L. et al., 2017; Giacomelli et al., 2020).

### Cellular to Non-cellular Patches

Cell-based cardiac patches have been designed to increase the survival ratio of the embedded cells and to ensure cellular retention (Braunwald, 2018; Zhu et al., 2021). For example, a recently developed cardiac patch fabricated with biomimetic microvessels and CSCs in fibrin gel enhanced angiogenesis, CSC retention, and survival rate after heart transplantation (Gao et al., 2018; Su et al., 2018a). Furthermore, non-cellular cardiac patches have been designed to overcome the limitations of live cellular cardiac patches (Tang et al., 2018b; Li Z. et al., 2021). For example, a specific miRNA patch was designed to modulate gene expression during cardiac remodeling (Wang L. L. et al., 2017). For miR-302 cardiac delivery, researchers designed a system by using a cholesterol molecule to decorate miR-302 mimics and using adamantane or cyclodextrin to modify hydrogels. Since the modified hydrogel showed self-assembly into shear-thinning and self-healing gels, the cholesterol on miR-302 mimics interacts with cyclodextrin to achieve sustained release (Wang L. L. et al., 2017; Huang et al., 2019a). To enhance clinical feasibility, an off-the-shelf cardiac patch was designed by embedding synthetic cardiac stromal cells into a decellularized extracellular matrix through a vacuum filtration method (Huang et al., 2020). In addition, exosomes derived from MSCs were integrated into hydrogels, providing a minimally invasive delivery method through intrapericardial injection. This injectable patch revolutionized the delivery of cardiac patches, which normally needs a traumatic open-chest surgery (Zhu and Cheng, 2021; Zhu et al., 2021).

### Tissue Patches

Cardiac tissue patch is one important type of cell-based patches that has been created by culture of embryonic stem cell-derived cardiomyocytes (ESC-CMs) (Chong et al., 2014), iPSC-CMs, or neonatal rodent cardiomyocytes (NRCMs) into a 3D scaffold to form a functional 3D structure (Pomeroy et al., 2020). For example, cardiac tissue patches cultured with multiple layers of iPSC-CMs or NRCMs within hydrogels or other porous polymer scaffolds form a randomly oriented and electromechanically integrated cardiac tissue patch (Shadrin et al., 2017; Pomeroy et al., 2020). In a window chamber model and a rodent MI model, the iPSC-CM 3D cardiac patch showed a preserved structure, electrical function, and successful vascularization (Shadrin et al., 2017; Cui et al., 2020). Implantation of this engineered cardiac tissue patch to the injured heart *in vivo* showed improved vascularization in the infarct region and reduced fibrosis (Wendel et al., 2014; Iseoka et al., 2018) (Uygun et al., 2010; Dvir et al., 2011; Fleischer et al., 2017).

3D bioprinting enables the production of 3D tissue constructs with precise architecture and integration of various cell types. The microenvironment of printed tissue accurately resembles native conditions, which, in turn, helps promote complex tissue formation *in vitro* (Gu et al., 2020). Different cellular techniques such as inkjet, stereolithography, and extrusion bioprinting are used for the development of cardiovascular tissues (Jana and Lerman, 2015; Duan, 2017). Common cell types used for cardiac tissue printing include MSCs, CSCs, ESCs, iPSCs, and cardiac fibroblasts (Cui et al., 2017). The laser-induced transfer (LIFT)-based cell bioprinting has been used to fabricate EC- and MSC-laden polyester urethane urea (PEUU) cardiac patches (Cui et al., 2018). When compared with non-patterned cardiac patches, patterned patches increased angiogenesis in the border zone of the infarction, as well as preservation of cardiac function after acute MI (Shengjie et al., 2009). 3D bioprinting could facilitate the development of the therapeutic potential of stem cells, which would play an important role in regenerative medicine (Wang et al., 2018; Pomeroy et al., 2020).

## Conclusion and Future Directions

Cellular reprogramming is a new paradigm in cell biology and provides a unique and efficient way to generate cell types of interest for cardiac repair by changing one cell fate to another (Wang et al., 2021). Usually, the indirect reprogramming routes require an *in vitro* engineered 3D tissue and then transplant *in vivo* (Querdel et al., 2021). The direct reprogramming bypass early developmental stages and administer the cardiac transcriptional factors directly by viral vectors. From a translational perspective, the technology of direct reprogramming holds great potential as a treatment due to its features including fast turnaround time and feasibility for *in vivo* applications (Wang et al., 2021). However, a large scale of somatic cells could be converted through indirect preprogramming to create a paradigm for *in vitro* CRISPR–Cas9 screening, drug screening, and disease modeling (Wang et al., 2021). Although cell reprogramming showed a potential strategy for the cardiac repair, there are few advantages and disadvantages in both reprogramming routes. Direct reprogramming rarely produces beating CMs after a long culture period (Fu et al., 2013; Nam et al., 2013). In comparison, indirect reprogramming is robust to produce beating CMs that are not in a mature stage. In addition, indirect reprogramming achieves a high conversion efficiency around 70 to even 90% (Pomeroy et al., 2020); however, direct reprogramming has a low conversion efficiency (4.8%) (Engel and Ardehali, 2018) due to the presence of epigenetic barriers such as Bmi1 (Zhou et al., 2016). This low conversion efficiency remains a major hurdle for direct reprogramming, and even the process was already improved by administering cardiac transcriptional factors along with epigenetic modifiers, inhibitors, cytokines, and miRNAs (Engel and Ardehali, 2018).

The goal of gene therapy for cardiac repair is to modify a gene or genetic pathway. Safety and efficacy are important to develop tolerance and ease administration that may be translated to the clinic (Wolfram and Donahue, 2013). For example, adenoviral vectors may trigger acute inflammation, which impacts gene transfer efficacy and may cause host morbidity (Liu and Muruve, 2003). Also, gene delivery through the myocardium or coronary injections has a low cardiac transfection outcome due to the neutralization by existing endogenous antibodies (Jessup et al., 2011). As previously mentioned, long-term expression of target genes may also cause sudden death in pig studies (Gabisonia et al., 2019).

Different types of stem cells have been studied as potential candidates for cardiac regenerative medicine. However, live stem cell delivery has many inherent limitations such as tumorigenicity, immunogenicity, cell death, and low retention after transplantation (Tang et al., 2018c). So, multiple cell-derived secretomes, exosomes, and miRNAs have been engineered as alternatives for heart repair by mimicking paracrine effects of the cell or manipulating gene expression during cardiac remodeling after MI. These cell-derived therapeutics have been combined with different biomaterials to overcome the limitations of low cardiac engraftment/retention, low miRNA stability, and delivery difficulties (Huang et al., 2019a).

Non-targeted cardiac therapeutics with an intravenous delivery usually affects multiple systems, which may cause systemic side effects. Targeted therapeutics, on the other hand, are designed for precise cardiac treatment with one or multiple intravenous injections. The targeting technology is achieved mainly through decorating a cardiac homing molecule on nanosized particles, which are safe in circulation with minimal chance of stimulating coagulations (Dobrovolskaia et al., 2009). Additionally, endogenous stem cells may be stimulated and redirected by BsAbs (Huang et al., 2019b; Liu M. et al., 2021). However, all targeting methods have to be further studied due to the low targeting capability and treatment efficacy.

3D cardiac patches are a promising method in cardiac repair and are either cellular or non-cellular. The cellular patches are generated through seeding of different live cells into various 3D scaffolds. To enhance the survival of the transplanted live cells, the patches have been engineered with mimetic blood vessels (Su et al., 2018a, 2020) or cocultured with ECs (Shadrin et al., 2017). For better integration, the scaffold has been engineered with microneedles (Tang et al., 2018b). Additionally, researchers have manipulated cell growth and differentiation conditions through culture medium optimization to enhance the maturity of iPSC-CMs on tissue patches (Machiraju and Greenway, 2019). Non-cellular patches are generated by seeding different cell derivatives into various 3D scaffolds. Compared with cellular cardiac patches, these patches have better stability, biocompatibility, modifiability, and low tumorigenicity and immunogenicity. 3D bioprinting technology has been widely utilized in cardiac repair by integrating biomaterials with different cell types to precisely pattern a cardiac structure (Liu N. et al., 2021). However, this technology is still in the early stage and needs to be improved (Liu N. et al., 2021).

Additional biotechnologies not mentioned in this review such as cardiac spheroids, single ventricles, bundles, regenerative gene expression, and design of biomaterials also play an important role in the field. Although there are plenty of technologies, it is not easy to get past the bottleneck of heart regenerative medicine, for example, the maturity of iPSC-CMs, the optimized cell protein factor combination, the detailed miRNA regulation mechanism, the key gene for cardiomyocyte regeneration, creation of large-sized cardiac tissue (Gao et al., 2018), and control of drug delivery-caused trauma. To overcome these obstacles, future interdisciplinary cooperation will be the key in the research area. For example, engineering of an injectable material that have controlled gelation speed, biocompatibility, degradative ability, and temperature sensitivity will be essential to create an injectable cardiac patch. Also, screening and designing of cardiotropic viral vectors through structure evolution of capsid variants (Tse et al., 2017) would enhance cardiac gene therapy. Moreover, natural exosomes are not clinically feasible due to many inherent limitations that could be overcome through cell-based pre-isolation exosome engineering and post-isolation exosome engineering (Huang P. et al., 2019; Jafari et al., 2020). Although mainly practiced in research labs today, innovative experimental bioengineering technologies will revolutionize heart repair field in the future.

## Author Contributions

MC, DZ, and KH wrote the text of this review article with guidance from KC and KH. All authors have reviewed the final version and approved the content in this manuscript.

## Conflict of Interest

The authors declare that the research was conducted in the absence of any commercial or financial relationships that could be construed as a potential conflict of interest.
